# Tetra­kis(1-ethyl-1*H*-imidazole-κ*N* ^3^)bis­(thio­cyanato-κ*N*)cadmium(II)

**DOI:** 10.1107/S1600536810004964

**Published:** 2010-02-13

**Authors:** Rong-Xun Li, Qi-Ye Wu, Fa-Qian Liu

**Affiliations:** a School of Materials Science and Engineering, University of Science and Technology Beijing, Beijing 100083, People’s Republic of China

## Abstract

The structure of the title compound, [Cd(NCS)_2_(C_5_H_8_N_2_)_4_], consists of isolated mol­ecules of [Cd(NCS)_2_(Eim)_4_] (Eim = 1-ethyl­imidazole), which contain a compressed octa­hedral CdN_6_ chromophore. The NCS^−^ anions are *trans* and four N atoms from the 1-ethyl­imidazole ligands define the equatorial plane. The mean Cd—N(Eim) and Cd—N(NCS) distances are 2.334 (4) and 2.379 (5) Å, respectively. Weak C—H⋯N inter­actions contribute to the crystal packing stability.

## Related literature

In the related cadmium compound [Cd(NCS)_2_(1-vinyl­imidazole)_4_], the Cd^II^ ions have a distorted octa­hedral environment, see: Liu *et al.* (2007 ▶).
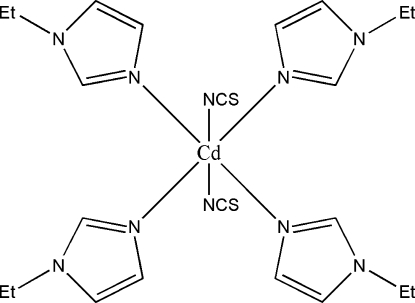


## Experimental

### 

#### Crystal data

[Cd(NCS)_2_(C_5_H_8_N_2_)_4_]
*M* *_r_* = 613.13
Triclinic, 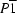

*a* = 9.0580 (18) Å
*b* = 13.532 (3) Å
*c* = 13.571 (3) Å
α = 69.45 (3)°
β = 70.88 (3)°
γ = 89.02 (3)°
*V* = 1462.6 (7) Å^3^
*Z* = 2
Mo *K*α radiation
μ = 0.92 mm^−1^
*T* = 293 K
0.30 × 0.30 × 0.20 mm

#### Data collection

Bruker SMART 1K CCD area-detector diffractometer
Absorption correction: multi-scan (*SADABS*; Sheldrick, 2004 ▶) *T*_min_ = 0.770, *T*_max_ = 0.838
6087 measured reflections
5708 independent reflections
4412 reflections with *I* > 2σ(*I*)
*R*_int_ = 0.024

#### Refinement

*R*[*F* ^2^ > 2σ(*F* ^2^)] = 0.049
*wR*(*F* ^2^) = 0.150
*S* = 1.00
5708 reflections
321 parameters
H-atom parameters constrained
Δρ_max_ = 0.87 e Å^−3^
Δρ_min_ = −0.85 e Å^−3^

### 

Data collection: *SMART* (Bruker, 2001 ▶); cell refinement: *SAINT* (Bruker, 2001 ▶); data reduction: *SAINT*; program(s) used to solve structure: *SHELXTL* (Sheldrick, 2008 ▶); program(s) used to refine structure: *SHELXTL*; molecular graphics: *SHELXTL*; software used to prepare material for publication: *SHELXTL* and local programs.

## Supplementary Material

Crystal structure: contains datablocks global, I. DOI: 10.1107/S1600536810004964/hg2644sup1.cif

Structure factors: contains datablocks I. DOI: 10.1107/S1600536810004964/hg2644Isup2.hkl

Additional supplementary materials:  crystallographic information; 3D view; checkCIF report

## Figures and Tables

**Table 1:** Hydrogen-bond geometry (Å, °)

*D*—H⋯*A*	*D*—H	H⋯*A*	*D*⋯*A*	*D*—H⋯*A*
C18—H18*A*⋯N1	0.93	2.81	3.324 (8)	116
C8—H8*A*⋯N2	0.93	2.72	3.279 (8)	119
C3—H3*A*⋯N5	0.93	2.97	3.346 (7)	106
C5—H5*A*⋯N1^i^	0.93	2.98	3.873 (8)	162

Symmetry code: (i) 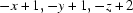.

**Fig. 1.**
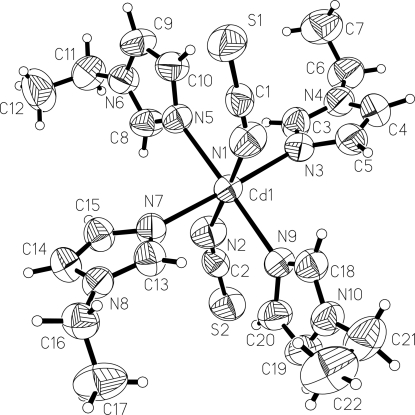
The structure of the title compound, showing 50% probability displacement ellipsoids and the atom-numbering scheme.

**Fig. 2.**
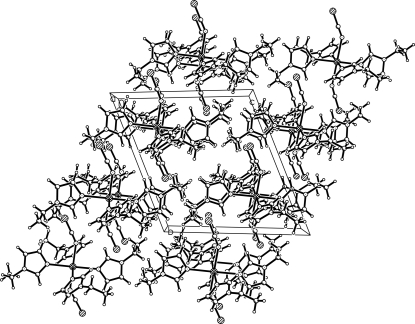
The packing of (I), viewed down the a axis.

## supplementary crystallographic information

### Comment

The molecular structure of (I) is shown in Fig. 1. The Cd atom displays an octahedral coordination geometry, with six N atoms from two thiocyanate anions and four 1-ethylimidazole ligands. The equatorial plane of the complex is formed by four Cd—*N*(1-ethylimadazole) bonds with lengths ranging from 2.331 (4) to 2.339 (4) Å, and the axial positions are occupied by two N-bonded NCS groups [Cd—N(NCS) = 2.369 (5) and 2.389 (4) Å]. These values agree well with those observed in [Cd(NCS)_2_(1-vinylimidazole)_4_] (Liu *et al.*, 2007). The values of the bond angles around cadmium are close to those expected for a regular octahedral geometry, the largest angular deviation being observed for the N3—Cd1—N9 angle [94.22 (12)°]. The thiocyanate ligands are almost linear. Weak C—H···N interactions contribute to the crystal packing stability.

In the corresponding cadmium compound [Cd(NCS)_2_(1-vinylimidazole)_4_] (Liu, *et al.*, 2007), the Cd^II^ ions have a distorted octahedral environment.

### Experimental

The title compound was prepared by the reaction of 1-ethylimidazole (1.92 g, 20 mmol) with CdCl_2_.0.5H_2_O(1.14 g, 5 mmol) and potassium thiocyanate (0.98 g, 10 mmol) by means of hydrothermal synthesis in stainless-steel reactor with Teflon liner at 383 K for 24 h. Single crystals suitable for X-ray measurements were obtained by recrystallization from methanol at room temperature.

### Refinement

H atoms were positioned geometrically(C—H = 0.93-0.97 Å) and allowed to ride on their parent atoms with U_iso_(H) = 1.2 times U_eq_(C).

### Crystal data

**Table:**

[Cd(NCS)_2_(C_5_H_8_N_2_)_4_]	*Z* = 2
*M**_r_* = 613.13	*F*(000) = 628
Triclinic, *P*1	*D*_x_ = 1.392 Mg m^−^^3^
Hall symbol: -P 1	Mo *K*α radiation, λ = 0.71073 Å
*a* = 9.0580 (18) Å	Cell parameters from 3229 reflections
*b* = 13.532 (3) Å	θ = 1.6–26.0°
*c* = 13.571 (3) Å	µ = 0.92 mm^−^^1^
α = 69.45 (3)°	*T* = 293 K
β = 70.88 (3)°	Block, colorless
γ = 89.02 (3)°	0.30 × 0.30 × 0.20 mm
*V* = 1462.6 (7) Å^3^	

### Data collection

**Table:**

Bruker SMART 1K CCD area-detector diffractometer	5708 independent reflections
Radiation source: fine-focus sealed tube	4412 reflections with *I* > 2σ(*I*)
graphite	*R*_int_ = 0.024
thin–slice ω scans	θ_max_ = 26.0°, θ_min_ = 1.6°
Absorption correction: multi-scan (*SADABS*; Sheldrick, 2004)	*h* = 0→11
*T*_min_ = 0.770, *T*_max_ = 0.838	*k* = −16→16
6087 measured reflections	*l* = −15→16

### Refinement

**Table:**

Refinement on *F*^2^	Secondary atom site location: difference Fourier map
Least-squares matrix: full	Hydrogen site location: inferred from neighbouring sites
*R*[*F*^2^ > 2σ(*F*^2^)] = 0.049	H-atom parameters constrained
*wR*(*F*^2^) = 0.150	*w* = 1/[σ^2^(*F*_o_^2^) + (0.1*P*)^2^] where *P* = (*F*_o_^2^ + 2*F*_c_^2^)/3
*S* = 1.00	(Δ/σ)_max_ = 0.001
5708 reflections	Δρ_max_ = 0.87 e Å^−^^3^
321 parameters	Δρ_min_ = −0.85 e Å^−^^3^
0 restraints	Extinction correction: *SHELXL*, Fc^*^=kFc[1+0.001xFc^2^λ^3^/sin(2θ)]^-1/4^
Primary atom site location: structure-invariant direct methods	Extinction coefficient: 0.038 (3)

### Special details

**Table:**

Geometry. All esds (except the esd in the dihedral angle between two l.s. planes) are estimated using the full covariance matrix. The cell esds are taken into account individually in the estimation of esds in distances, angles and torsion angles; correlations between esds in cell parameters are only used when they are defined by crystal symmetry. An approximate (isotropic) treatment of cell esds is used for estimating esds involving l.s. planes.
Refinement. Refinement of *F*^2^ against ALL reflections. The weighted *R*-factor wR and goodness of fit *S* are based on *F*^2^, conventional *R*-factors *R* are based on *F*, with *F* set to zero for negative *F*^2^. The threshold expression of *F*^2^ > σ(*F*^2^) is used only for calculating *R*-factors(gt) etc. and is not relevant to the choice of reflections for refinement. *R*-factors based on *F*^2^ are statistically about twice as large as those based on *F*, and *R*- factors based on ALL data will be even larger.

### Fractional atomic coordinates and isotropic or equivalent isotropic displacement parameters (Å^2^)

**Table:**

	*x*	*y*	*z*	*U*_iso_*/*U*_eq_	
Cd1	0.69564 (4)	0.74519 (2)	0.74788 (3)	0.05176 (18)	
S1	0.8813 (2)	0.38753 (12)	0.83690 (15)	0.0894 (5)	
S2	0.5096 (2)	1.10420 (12)	0.64503 (16)	0.0908 (5)	
N7	0.9399 (5)	0.8103 (3)	0.7377 (3)	0.0619 (10)	
N3	0.4588 (5)	0.6765 (3)	0.7552 (3)	0.0598 (10)	
N5	0.8071 (5)	0.7265 (3)	0.5749 (3)	0.0589 (9)	
N8	1.1286 (5)	0.8430 (3)	0.7960 (3)	0.0577 (9)	
N4	0.2773 (5)	0.6417 (4)	0.6931 (4)	0.0695 (11)	
N1	0.7430 (6)	0.5694 (4)	0.8445 (4)	0.0744 (12)	
C1	0.7999 (5)	0.4933 (4)	0.8406 (4)	0.0549 (10)	
N9	0.5902 (5)	0.7659 (3)	0.9200 (3)	0.0600 (9)	
N2	0.6550 (6)	0.9207 (4)	0.6501 (4)	0.0780 (12)	
C2	0.5964 (6)	0.9977 (4)	0.6467 (4)	0.0578 (11)	
C13	0.9874 (6)	0.7894 (4)	0.8245 (4)	0.0640 (12)	
H13A	0.9304	0.7433	0.8966	0.077*	
N6	0.9110 (5)	0.7726 (3)	0.3931 (3)	0.0664 (11)	
C20	0.5788 (7)	0.8579 (4)	0.9399 (5)	0.0712 (13)	
H20A	0.5914	0.9255	0.8858	0.085*	
C15	1.0593 (6)	0.8803 (4)	0.6491 (4)	0.0647 (12)	
H15A	1.0593	0.9107	0.5761	0.078*	
C18	0.5643 (6)	0.6902 (4)	1.0195 (4)	0.0661 (12)	
H18A	0.5654	0.6183	1.0313	0.079*	
C8	0.8485 (7)	0.8045 (4)	0.4788 (4)	0.0689 (13)	
H8A	0.8359	0.8749	0.4707	0.083*	
C10	0.8447 (7)	0.6377 (4)	0.5496 (4)	0.0682 (13)	
H10A	0.8278	0.5687	0.6017	0.082*	
C5	0.3418 (7)	0.6026 (4)	0.8429 (5)	0.0692 (13)	
H5A	0.3398	0.5730	0.9164	0.083*	
C14	1.1764 (6)	0.8981 (4)	0.6839 (4)	0.0668 (13)	
H14A	1.2716	0.9401	0.6396	0.080*	
C9	0.9098 (7)	0.6655 (4)	0.4379 (4)	0.0740 (15)	
H9A	0.9465	0.6205	0.3990	0.089*	
C4	0.2306 (7)	0.5801 (4)	0.8049 (5)	0.0761 (15)	
H4A	0.1404	0.5323	0.8465	0.091*	
C3	0.4137 (6)	0.6964 (4)	0.6684 (4)	0.0676 (13)	
H3A	0.4709	0.7437	0.5970	0.081*	
N10	0.5365 (5)	0.7290 (4)	1.1004 (3)	0.0689 (11)	
C16	1.2126 (7)	0.8384 (5)	0.8732 (5)	0.0717 (14)	
H16A	1.2021	0.7656	0.9242	0.086*	
H16B	1.3235	0.8603	0.8305	0.086*	
C6	0.1968 (8)	0.6455 (6)	0.6151 (6)	0.094 (2)	
H6A	0.0907	0.6104	0.6573	0.113*	
H6B	0.1893	0.7191	0.5738	0.113*	
C19	0.5463 (7)	0.8359 (5)	1.0506 (5)	0.0770 (15)	
H19A	0.5332	0.8849	1.0858	0.092*	
C11	0.9731 (8)	0.8412 (5)	0.2743 (4)	0.0855 (18)	
H11A	0.9749	0.7991	0.2291	0.103*	
H11B	0.9032	0.8953	0.2593	0.103*	
C7	0.2747 (9)	0.5958 (6)	0.5366 (6)	0.112 (2)	
H7A	0.2365	0.6183	0.4749	0.168*	
H7B	0.2535	0.5200	0.5733	0.168*	
H7C	0.3860	0.6159	0.5094	0.168*	
C17	1.1544 (10)	0.9058 (6)	0.9382 (6)	0.108 (2)	
H17A	1.1984	0.8898	0.9968	0.162*	
H17B	1.0418	0.8928	0.9706	0.162*	
H17C	1.1846	0.9790	0.8901	0.162*	
C21	0.5084 (9)	0.6655 (6)	1.2194 (5)	0.101 (2)	
H21A	0.4195	0.6886	1.2659	0.121*	
H21B	0.4825	0.5914	1.2336	0.121*	
C22	0.6465 (10)	0.6764 (7)	1.2495 (7)	0.124 (3)	
H22A	0.6231	0.6372	1.3280	0.186*	
H22B	0.6743	0.7500	1.2332	0.186*	
H22C	0.7327	0.6492	1.2071	0.186*	
C12	1.1279 (9)	0.8917 (6)	0.2418 (6)	0.128 (3)	
H12A	1.1616	0.9374	0.1643	0.192*	
H12B	1.1989	0.8387	0.2524	0.192*	
H12C	1.1272	0.9329	0.2868	0.192*	

### Atomic displacement parameters (Å^2^)

**Table:**

	*U*^11^	*U*^22^	*U*^33^	*U*^12^	*U*^13^	*U*^23^
Cd1	0.0538 (2)	0.0523 (2)	0.0494 (2)	0.00817 (14)	−0.01671 (15)	−0.01999 (15)
S1	0.0938 (11)	0.0607 (8)	0.0959 (11)	0.0180 (8)	−0.0099 (9)	−0.0294 (8)
S2	0.1011 (12)	0.0581 (8)	0.1125 (12)	0.0222 (8)	−0.0405 (10)	−0.0274 (8)
N7	0.056 (2)	0.067 (2)	0.060 (2)	0.0033 (19)	−0.0172 (19)	−0.022 (2)
N3	0.060 (2)	0.061 (2)	0.060 (2)	0.0050 (19)	−0.0202 (19)	−0.0252 (19)
N5	0.065 (2)	0.061 (2)	0.051 (2)	0.0095 (19)	−0.0187 (18)	−0.0218 (18)
N8	0.055 (2)	0.064 (2)	0.056 (2)	0.0100 (18)	−0.0183 (18)	−0.0246 (19)
N4	0.059 (2)	0.088 (3)	0.074 (3)	0.008 (2)	−0.025 (2)	−0.042 (2)
N1	0.083 (3)	0.067 (3)	0.068 (3)	0.023 (2)	−0.024 (2)	−0.021 (2)
C1	0.054 (3)	0.051 (3)	0.046 (2)	−0.002 (2)	−0.0098 (19)	−0.0088 (19)
N9	0.059 (2)	0.065 (2)	0.057 (2)	0.0081 (18)	−0.0157 (18)	−0.0280 (19)
N2	0.092 (3)	0.064 (3)	0.080 (3)	0.023 (2)	−0.036 (3)	−0.024 (2)
C2	0.065 (3)	0.050 (3)	0.051 (2)	−0.002 (2)	−0.021 (2)	−0.008 (2)
C13	0.062 (3)	0.069 (3)	0.057 (3)	0.003 (2)	−0.021 (2)	−0.016 (2)
N6	0.081 (3)	0.064 (3)	0.051 (2)	0.000 (2)	−0.019 (2)	−0.0190 (19)
C20	0.081 (4)	0.064 (3)	0.070 (3)	0.010 (3)	−0.020 (3)	−0.030 (3)
C15	0.065 (3)	0.064 (3)	0.057 (3)	−0.001 (2)	−0.020 (2)	−0.013 (2)
C18	0.070 (3)	0.069 (3)	0.060 (3)	0.008 (2)	−0.022 (2)	−0.024 (3)
C8	0.096 (4)	0.054 (3)	0.056 (3)	0.010 (3)	−0.025 (3)	−0.021 (2)
C10	0.085 (4)	0.059 (3)	0.060 (3)	0.003 (3)	−0.023 (3)	−0.022 (2)
C5	0.075 (3)	0.061 (3)	0.068 (3)	0.005 (3)	−0.021 (3)	−0.021 (2)
C14	0.062 (3)	0.063 (3)	0.069 (3)	−0.004 (2)	−0.022 (3)	−0.017 (2)
C9	0.094 (4)	0.074 (4)	0.064 (3)	0.017 (3)	−0.026 (3)	−0.038 (3)
C4	0.069 (3)	0.071 (3)	0.080 (4)	−0.004 (3)	−0.018 (3)	−0.025 (3)
C3	0.056 (3)	0.083 (4)	0.063 (3)	0.004 (3)	−0.020 (2)	−0.028 (3)
N10	0.068 (3)	0.086 (3)	0.054 (2)	0.006 (2)	−0.019 (2)	−0.028 (2)
C16	0.067 (3)	0.079 (4)	0.083 (4)	0.014 (3)	−0.039 (3)	−0.034 (3)
C6	0.078 (4)	0.134 (6)	0.104 (5)	0.023 (4)	−0.046 (4)	−0.070 (5)
C19	0.086 (4)	0.082 (4)	0.074 (4)	0.015 (3)	−0.024 (3)	−0.044 (3)
C11	0.101 (5)	0.097 (4)	0.048 (3)	0.001 (4)	−0.019 (3)	−0.021 (3)
C7	0.123 (6)	0.141 (7)	0.108 (5)	0.023 (5)	−0.056 (5)	−0.072 (5)
C17	0.141 (7)	0.128 (6)	0.101 (5)	0.049 (5)	−0.075 (5)	−0.066 (5)
C21	0.114 (6)	0.134 (6)	0.051 (3)	0.016 (5)	−0.027 (3)	−0.030 (4)
C22	0.134 (7)	0.160 (8)	0.119 (6)	0.065 (6)	−0.075 (6)	−0.071 (6)
C12	0.121 (6)	0.144 (7)	0.087 (5)	−0.055 (5)	−0.006 (4)	−0.028 (5)

### Geometric parameters (Å, °)

**Table:**

Cd1—N3	2.311 (4)	C10—C9	1.345 (7)
Cd1—N9	2.331 (4)	C10—H10A	0.9300
Cd1—N5	2.334 (4)	C5—C4	1.354 (8)
Cd1—N7	2.339 (4)	C5—H5A	0.9300
Cd1—N2	2.370 (5)	C14—H14A	0.9300
Cd1—N1	2.389 (4)	C9—H9A	0.9300
S1—C1	1.608 (5)	C4—H4A	0.9300
S2—C2	1.627 (5)	C3—H3A	0.9300
N7—C13	1.322 (6)	N10—C19	1.356 (7)
N7—C15	1.377 (6)	N10—C21	1.474 (7)
N3—C3	1.310 (6)	C16—C17	1.460 (8)
N3—C5	1.383 (7)	C16—H16A	0.9700
N5—C8	1.296 (6)	C16—H16B	0.9700
N5—C10	1.371 (6)	C6—C7	1.442 (8)
N8—C13	1.350 (6)	C6—H6A	0.9700
N8—C14	1.357 (6)	C6—H6B	0.9700
N8—C16	1.467 (6)	C19—H19A	0.9300
N4—C3	1.331 (7)	C11—C12	1.429 (9)
N4—C4	1.371 (7)	C11—H11A	0.9700
N4—C6	1.457 (7)	C11—H11B	0.9700
N1—C1	1.154 (6)	C7—H7A	0.9600
N9—C18	1.326 (6)	C7—H7B	0.9600
N9—C20	1.359 (6)	C7—H7C	0.9600
N2—C2	1.154 (6)	C17—H17A	0.9600
C13—H13A	0.9300	C17—H17B	0.9600
N6—C8	1.332 (6)	C17—H17C	0.9600
N6—C9	1.359 (6)	C21—C22	1.462 (10)
N6—C11	1.467 (6)	C21—H21A	0.9700
C20—C19	1.354 (7)	C21—H21B	0.9700
C20—H20A	0.9300	C22—H22A	0.9600
C15—C14	1.350 (7)	C22—H22B	0.9600
C15—H15A	0.9300	C22—H22C	0.9600
C18—N10	1.328 (6)	C12—H12A	0.9600
C18—H18A	0.9300	C12—H12B	0.9600
C8—H8A	0.9300	C12—H12C	0.9600
			
N3—Cd1—N9	94.24 (14)	C10—C9—N6	106.3 (5)
N3—Cd1—N5	87.15 (14)	C10—C9—H9A	126.8
N9—Cd1—N5	178.61 (13)	N6—C9—H9A	126.8
N3—Cd1—N7	177.92 (13)	C5—C4—N4	106.5 (5)
N9—Cd1—N7	87.36 (14)	C5—C4—H4A	126.8
N5—Cd1—N7	91.26 (14)	N4—C4—H4A	126.8
N3—Cd1—N2	92.07 (17)	N3—C3—N4	112.9 (5)
N9—Cd1—N2	91.78 (16)	N3—C3—H3A	123.5
N5—Cd1—N2	88.03 (16)	N4—C3—H3A	123.5
N7—Cd1—N2	89.21 (17)	C18—N10—C19	106.4 (4)
N3—Cd1—N1	89.13 (16)	C18—N10—C21	125.2 (5)
N9—Cd1—N1	88.71 (15)	C19—N10—C21	128.3 (5)
N5—Cd1—N1	91.45 (15)	C17—C16—N8	112.9 (5)
N7—Cd1—N1	89.58 (16)	C17—C16—H16A	109.0
N2—Cd1—N1	178.67 (16)	N8—C16—H16A	109.0
C13—N7—C15	104.6 (4)	C17—C16—H16B	109.0
C13—N7—Cd1	124.5 (3)	N8—C16—H16B	109.0
C15—N7—Cd1	130.9 (3)	H16A—C16—H16B	107.8
C3—N3—C5	104.7 (4)	C7—C6—N4	113.2 (6)
C3—N3—Cd1	124.5 (3)	C7—C6—H6A	108.9
C5—N3—Cd1	130.6 (4)	N4—C6—H6A	108.9
C8—N5—C10	104.9 (4)	C7—C6—H6B	108.9
C8—N5—Cd1	124.6 (3)	N4—C6—H6B	108.9
C10—N5—Cd1	130.5 (3)	H6A—C6—H6B	107.7
C13—N8—C14	106.6 (4)	C20—C19—N10	107.0 (5)
C13—N8—C16	125.4 (4)	C20—C19—H19A	126.5
C14—N8—C16	128.0 (4)	N10—C19—H19A	126.5
C3—N4—C4	106.5 (4)	C12—C11—N6	112.7 (6)
C3—N4—C6	126.2 (5)	C12—C11—H11A	109.0
C4—N4—C6	127.3 (5)	N6—C11—H11A	109.0
C1—N1—Cd1	148.8 (4)	C12—C11—H11B	109.0
N1—C1—S1	178.7 (5)	N6—C11—H11B	109.0
C18—N9—C20	104.9 (4)	H11A—C11—H11B	107.8
C18—N9—Cd1	125.6 (3)	C6—C7—H7A	109.5
C20—N9—Cd1	127.8 (3)	C6—C7—H7B	109.5
C2—N2—Cd1	152.1 (4)	H7A—C7—H7B	109.5
N2—C2—S2	178.3 (5)	C6—C7—H7C	109.5
N7—C13—N8	112.0 (4)	H7A—C7—H7C	109.5
N7—C13—H13A	124.0	H7B—C7—H7C	109.5
N8—C13—H13A	124.0	C16—C17—H17A	109.5
C8—N6—C9	106.5 (4)	C16—C17—H17B	109.5
C8—N6—C11	126.1 (5)	H17A—C17—H17B	109.5
C9—N6—C11	127.4 (5)	C16—C17—H17C	109.5
C19—C20—N9	109.4 (5)	H17A—C17—H17C	109.5
C19—C20—H20A	125.3	H17B—C17—H17C	109.5
N9—C20—H20A	125.3	C22—C21—N10	111.3 (7)
C14—C15—N7	109.9 (5)	C22—C21—H21A	109.4
C14—C15—H15A	125.0	N10—C21—H21A	109.4
N7—C15—H15A	125.0	C22—C21—H21B	109.4
N9—C18—N10	112.2 (5)	N10—C21—H21B	109.4
N9—C18—H18A	123.9	H21A—C21—H21B	108.0
N10—C18—H18A	123.9	C21—C22—H22A	109.5
N5—C8—N6	112.6 (4)	C21—C22—H22B	109.5
N5—C8—H8A	123.7	H22A—C22—H22B	109.5
N6—C8—H8A	123.7	C21—C22—H22C	109.5
C9—C10—N5	109.6 (5)	H22A—C22—H22C	109.5
C9—C10—H10A	125.2	H22B—C22—H22C	109.5
N5—C10—H10A	125.2	C11—C12—H12A	109.5
C4—C5—N3	109.4 (5)	C11—C12—H12B	109.5
C4—C5—H5A	125.3	H12A—C12—H12B	109.5
N3—C5—H5A	125.3	C11—C12—H12C	109.5
C15—C14—N8	106.9 (5)	H12A—C12—H12C	109.5
C15—C14—H14A	126.6	H12B—C12—H12C	109.5
N8—C14—H14A	126.6		
			
N3—Cd1—N7—C13	−103 (4)	N5—Cd1—N2—C2	163.7 (10)
N9—Cd1—N7—C13	36.8 (4)	N7—Cd1—N2—C2	−105.1 (10)
N5—Cd1—N7—C13	−143.4 (4)	N1—Cd1—N2—C2	−129 (6)
N2—Cd1—N7—C13	128.6 (4)	Cd1—N2—C2—S2	−23 (18)
N1—Cd1—N7—C13	−51.9 (4)	C15—N7—C13—N8	0.7 (6)
N3—Cd1—N7—C15	81 (4)	Cd1—N7—C13—N8	−175.8 (3)
N9—Cd1—N7—C15	−138.7 (4)	C14—N8—C13—N7	−2.3 (6)
N5—Cd1—N7—C15	41.1 (4)	C16—N8—C13—N7	179.1 (4)
N2—Cd1—N7—C15	−46.9 (4)	C18—N9—C20—C19	0.0 (6)
N1—Cd1—N7—C15	132.6 (4)	Cd1—N9—C20—C19	−165.4 (4)
N9—Cd1—N3—C3	142.9 (4)	C13—N7—C15—C14	1.2 (6)
N5—Cd1—N3—C3	−36.9 (4)	Cd1—N7—C15—C14	177.4 (3)
N7—Cd1—N3—C3	−77 (4)	C20—N9—C18—N10	0.4 (6)
N2—Cd1—N3—C3	51.0 (4)	Cd1—N9—C18—N10	166.2 (3)
N1—Cd1—N3—C3	−128.4 (4)	C10—N5—C8—N6	−0.4 (6)
N9—Cd1—N3—C5	−42.8 (4)	Cd1—N5—C8—N6	179.1 (3)
N5—Cd1—N3—C5	137.4 (4)	C9—N6—C8—N5	−0.1 (7)
N7—Cd1—N3—C5	97 (4)	C11—N6—C8—N5	−178.3 (5)
N2—Cd1—N3—C5	−134.7 (4)	C8—N5—C10—C9	0.7 (6)
N1—Cd1—N3—C5	45.9 (4)	Cd1—N5—C10—C9	−178.8 (4)
N3—Cd1—N5—C8	105.4 (4)	C3—N3—C5—C4	0.5 (6)
N9—Cd1—N5—C8	−69 (5)	Cd1—N3—C5—C4	−174.7 (4)
N7—Cd1—N5—C8	−76.0 (4)	N7—C15—C14—N8	−2.6 (6)
N2—Cd1—N5—C8	13.2 (4)	C13—N8—C14—C15	3.0 (6)
N1—Cd1—N5—C8	−165.6 (4)	C16—N8—C14—C15	−178.6 (5)
N3—Cd1—N5—C10	−75.3 (5)	N5—C10—C9—N6	−0.7 (7)
N9—Cd1—N5—C10	111 (5)	C8—N6—C9—C10	0.5 (6)
N7—Cd1—N5—C10	103.4 (5)	C11—N6—C9—C10	178.7 (5)
N2—Cd1—N5—C10	−167.4 (5)	N3—C5—C4—N4	−1.0 (6)
N1—Cd1—N5—C10	13.8 (5)	C3—N4—C4—C5	1.0 (6)
N3—Cd1—N1—C1	95.1 (8)	C6—N4—C4—C5	179.7 (5)
N9—Cd1—N1—C1	−170.6 (9)	C5—N3—C3—N4	0.2 (6)
N5—Cd1—N1—C1	8.0 (9)	Cd1—N3—C3—N4	175.7 (3)
N7—Cd1—N1—C1	−83.3 (8)	C4—N4—C3—N3	−0.8 (6)
N2—Cd1—N1—C1	−59 (6)	C6—N4—C3—N3	−179.4 (5)
Cd1—N1—C1—S1	122 (22)	N9—C18—N10—C19	−0.6 (6)
N3—Cd1—N9—C18	82.4 (4)	N9—C18—N10—C21	−178.0 (5)
N5—Cd1—N9—C18	−103 (5)	C13—N8—C16—C17	−80.3 (7)
N7—Cd1—N9—C18	−96.3 (4)	C14—N8—C16—C17	101.5 (7)
N2—Cd1—N9—C18	174.6 (4)	C3—N4—C6—C7	70.8 (9)
N1—Cd1—N9—C18	−6.7 (4)	C4—N4—C6—C7	−107.5 (7)
N3—Cd1—N9—C20	−115.1 (4)	N9—C20—C19—N10	−0.3 (7)
N5—Cd1—N9—C20	59 (5)	C18—N10—C19—C20	0.5 (6)
N7—Cd1—N9—C20	66.2 (4)	C21—N10—C19—C20	177.9 (6)
N2—Cd1—N9—C20	−22.9 (4)	C8—N6—C11—C12	79.8 (9)
N1—Cd1—N9—C20	155.9 (4)	C9—N6—C11—C12	−98.2 (8)
N3—Cd1—N2—C2	76.6 (10)	C18—N10—C21—C22	104.3 (7)
N9—Cd1—N2—C2	−17.7 (10)	C19—N10—C21—C22	−72.6 (9)

### Hydrogen-bond geometry (Å, °)

**Table:**

*D*—H···*A*	*D*—H	H···*A*	*D*···*A*	*D*—H···*A*
C18—H18A···N1	0.93	2.81	3.324 (8)	116
C8—H8A···N2	0.93	2.72	3.279 (8)	119
C3—H3A···N5	0.93	2.97	3.346 (7)	106
C5—H5A···N1^i^	0.93	2.98	3.873 (8)	162

Symmetry codes: (i) −*x*+1, −*y*+1, −*z*+2.
